# Ruthenium Complexes With Piplartine Cause Apoptosis Through MAPK Signaling by a p53-Dependent Pathway in Human Colon Carcinoma Cells and Inhibit Tumor Development in a Xenograft Model

**DOI:** 10.3389/fonc.2019.00582

**Published:** 2019-07-03

**Authors:** Ingrid R. S. Baliza, Suellen L. R. Silva, Luciano de S. Santos, João H. Araujo Neto, Rosane B. Dias, Caroline B. S. Sales, Clarissa A. Gurgel Rocha, Milena B. P. Soares, Alzir A. Batista, Daniel P. Bezerra

**Affiliations:** ^1^Gonçalo Moniz Institute, Oswaldo Cruz Foundation (IGM-FIOCRUZ/BA), Salvador, Brazil; ^2^Department of Chemistry, Federal University of São Carlos, São Carlos, Brazil; ^3^Department of Biomorphology, Institute of Health Sciences, Federal University of Bahia, Salvador, Brazil

**Keywords:** ruthenium complexes, piplartine, piperlongumine, apoptosis, MAPK, p53

## Abstract

Ruthenium complexes with piplartine, [Ru(piplartine)(dppf)(bipy)](PF_6_)_2_ (**1**) and [Ru(piplartine)(dppb)(bipy)](PF_6_)_2_ (**2**) (dppf = 1,1-bis(diphenylphosphino) ferrocene; dppb = 1,4-bis(diphenylphosphino)butane and bipy = 2,2′-bipyridine), were recently synthesized and displayed more potent cytotoxicity than piplartine in different cancer cells, regulated RNA transcripts of several apoptosis-related genes, and induced reactive oxygen species (ROS)-mediated apoptosis in human colon carcinoma HCT116 cells. The present work aimed to explore the underlying mechanisms through which these ruthenium complexes induce cell death in HCT116 cells *in vitro*, as well as their *in vivo* action in a xenograft model. Both complexes significantly increased the percentage of apoptotic HCT116 cells, and co-treatment with inhibitors of JNK/SAPK, p38 MAPK, and MEK, which inhibits the activation of ERK1/2, significantly reduced the apoptosis rate induced by these complexes. Moreover, significant increase in phospho-JNK2 (T183/Y185), phospho-p38α (T180/Y182), and phospho-ERK1 (T202/Y204) expressions were observed in cells treated with these complexes, indicating MAPK-mediated apoptosis. In addition, co-treatment with a p53 inhibitor (cyclic pifithrin-α) and the ruthenium complexes significantly reduced the apoptosis rate in HCT116 cells, and increased phospho-p53 (S15) and phospho-histone H2AX (S139) expressions, indicating induction of DNA damage and p53-dependent apoptosis. Both complexes also reduced HCT116 cell growth in a xenograft model. Tumor mass inhibition rates were 35.06, 29.71, and 32.03% for the complex **1** (15 μmol/kg/day), complex **2** (15 μmol/kg/day), and piplartine (60 μmol/kg/day), respectively. These data indicate these ruthenium complexes as new anti-colon cancer drugs candidates.

## Introduction

Colorectal cancer (CRC) is a lethal disease that ranks third in incidence and second in mortality. In 2018, 1.8 million new CRC cases and 881,000 deaths were estimated to occur worldwide (1). Currently, cytotoxic chemotherapy with FOLFOX (leucovorin, 5-fluorouracil, and oxaliplatin), FOLFIRI (leucovorin, 5-fluorouracil, and irinotecan), or FOLFOXIRI (leucovorin, 5-fluorouracil, oxaliplatin, and irinotecan) are standard regimens most often used (2). However, CRC remains with a high mortality and new treatment strategies are urgently needed.

Piplartine (piperlongumine) is a natural molecule found in *Piper* species that has multiple pharmacological effects. In particular, potent cytotoxic, genotoxic, antitumor, antiangiogenic, and antimetastatic properties, along with favorable pharmacokinetic profile and safety has been attributed to this molecule. Therefore, piplartine and its analogs have been considered prototypes for the development of new antineoplastic agents (3–17). The mechanism of the cell death induced by this molecule include elevation of the intracellular levels of reactive oxygen species (ROS) selectively in cancer cells, activation of MAPK and p53 signaling and inhibition of NFκB pathway (10, 11, 15, 18–21).

Ruthenium complexes are a potential class of antineoplastic agents, being their antitumor effect dependent on the structure of the ligands bound to the metal. Currently, some ruthenium complexes with different ligands with potent cancer cell death activity are under pre-clinical development or phase I/II clinical trials (22–30). Among them, ruthenium complexes with xanthoxylin, thymine, p-cymene, and/or 5-fluorouracil ligands are currently under pre-clinical development with promising results (24–30). A clinical trial phase I dose-escalation study was concluded with ruthenium complex indazolium trans-[tetrachlorobis(1H-indazole)ruthenate(III)] (KP1019, FFC14A) in patients with different types of solid tumors and clinical trial phase I/II study with imidazolium-trans-tetrachloro(dimethylsulfoxide) imidazoleruthenium(III) (NAMI-A) plus gemcitabine was performed in patients with non-small cell lung cancer (22, 23). Their mechanisms of action include DNA interaction, cell cycle arrest, induction of oxidative stress and apoptotic cell death (24–30).

**Figure 1:**
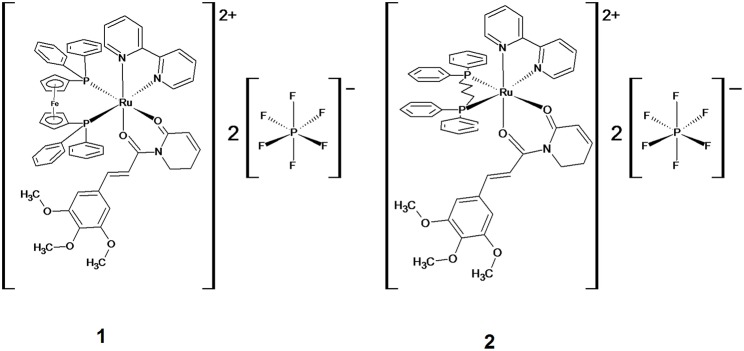
Chemical structure of ruthenium complexes with piplartine, [Ru(piplartine)(dppf)(bipy)](PF_6_)_2_ (**1**) and [Ru(piplartine)(dppb)(bipy)](PF_6_)_2_ (**2**).

Recently, we designed and synthesized two novel ruthenium complexes with piplartine (Figure 1), [Ru(piplartine)(dppf)(bipy)](PF_6_)_2_ (**1**) and [Ru(piplartine)(dppb)(bipy)](PF_6_)_2_ (**2**) (dppf = 1,1-bis(diphenylphosphino) ferrocene; dppb = 1,4-bis(diphenylphosphino)butane and bipy = 2,2′-bipyridine), that displayed cytotoxicity more potent than piplartine in a panel of eight different cancer cells, with less effect in non-cancer cells. Additionally, we showed that their apoptosis-inducing effect was mediated by ROS in human colon carcinoma HCT116 cells, which was accompanied by up-regulated expression of some MAPK- and p53-related genes (31). The present work aimed to explore the underlying mechanisms by which these ruthenium complexes induce cell death in HCT116 cells *in vitro*, as well as their *in vivo* action in a xenograft model.

## Materials and Methods

### Synthesis of the Ruthenium Complexes With Piplartine

Ruthenium complexes with piplartine [Ru(piplartine)(dppf)(bipy)](PF_6_)_2_ (**1**) and [Ru(piplartine)(dppb)(bipy)](PF_6_)_2_ (**2**) were obtained using two different precursors, [RuCl_2_(N-N)(P-P)] (N-N = 2,2′-bipyridine (bipy); P-P = 1,1′-bis(diphenylphosphino)ferrocene (dppf) for complex **1** (heterometallic), and 1,4-bis(diphenylphosphino)butane (dppb) for complex **2** (monometallic), as previously described by D'Sousa Costa et al. (31). All procedures involving solutions of complexes were performed under inert atmosphere (argon). Solvents used in manipulations were purified by standard methods. Piplartine was purchased from Cayman Chemical Company (Cayman Chemical, Ann Arbor, MI, USA) and all other reagents were purchased from Sigma-Aldrich (Sigma-Aldrich Co., Saint Louis, MO, USA).

### *In vitro* Assays

#### Cells

Human colon carcinoma HCT116 cells were obtained from the American Type Culture Collection (ATCC, Manassas, VA, USA). Cells were cultured as recommended by ATCC and a mycoplasma stain kit (Sigma-Aldrich) was used to validate the use of cells free from contamination. Cell viability in all experiments was examined using the trypan blue exclusion assay. Over 90% of the cells were viable at the beginning of the culture.

#### Apoptosis Quantification Assay

FITC Annexin V Apoptosis Detection Kit I (BD Biosciences) was used for apoptosis quantification, and the analysis was performed according to the manufacturer's instructions. Cell fluorescence was determined by flow cytometry. At least 10^4^ events were recorded per sample using a BD LSRFortessa cytometer, BD FACSDiva Software (BD Biosciences) and FlowJo Software 10 (FlowJo Lcc; Ashland, OR, USA). Cellular debris were omitted from the analysis. Percentages of viable, early apoptotic, late apoptotic and necrotic cells were determined. Protection assays using a JNK/SAPK inhibitor (SP600125; Cayman Chemical), p38 MAPK inhibitor (PD169316; Cayman Chemical), MEK (mitogen-activated protein kinase kinase) inhibitor (U0126; Cayman Chemical), and p53 inhibitor (cyclic pifithrin-α; Cayman Chemical) were performed. In these assays, cells were pre-incubated for 2 h with 5 μM SP600125, 5 μM PD169316, 5 μM U0126, or 10 μM cyclic pifithrin-α, followed by incubation with complexes at previously established concentrations (2.5 μM for complex **1** and 5 μM for complex **2**) for 48 h (31). Negative control was treated with the vehicle (0.1% of a solution containing 70% sorbitol, 25% tween 80 and 5% water) used for diluting the compounds tested. Doxorubicin (1 μM) and piplartine (10 μM) were used as positive controls.

#### Phospho-Specific ELISA

Human phospho-JNK2 (T183/Y185), phospho-p38α (T180/Y182), phospho-ERK1 (T202/Y204), phospho-p53 (S15), total MDM2 and phospho-histone H2AX (S139) were quantified in cell lysates using sandwich ELISA kits (R&D Systems, Inc. Minneapolis, MN, USA), and the analysis was performed according to the manufacturer's instructions. Cells were lysed in a buffer solution containing 100 mM tris, pH 7.4, 150 mM NaCl, 1 mM EGTA, 1 mM EDTA, 1% triton X-100, and 0.5% sodium deoxycholate plus phosphatase inhibitor cocktail, protease inhibitor cocktail and 1 mM PMSF immediately before use (all purchased from Sigma-Aldrich Co.). Total protein quantification was performed in each sample by Pierce Protein Assay (Thermo Fisher Scientific, Waltham, MA, USA) using BSA as standard. Absorbance at 450 nm was measured using the SpectraMax 190 Microplate Reader (Molecular Devices, Sunnyvale, CA, USA).

### *In vivo* Assays

#### Animals

A total of 41 Specific Pathogen-Free (SPF) C.B*-*17 SCID mice (females, 6–8 weeks, 20–25 g) was obtained and maintained at the animal facilities from Gonçalo Moniz Institute-FIOCRUZ (Salvador, Bahia, Brazil). Animals were housed in cages with free access to food and water. All animals were kept under a 12:12 h light-dark cycle (lights on at 6:00 a.m.). The institutional Animal Ethics Committee of Gonçalo Moniz Institute (Fiocruz-BA/Brazil) approved the experimental protocol (number 06/2015). Animal welfare was monitored throughout the study, and the pain and suffering were minimized.

#### Human Colon Carcinoma Xenograft Model

HCT116 cells (10^7^ cells per 500 μL) were implanted subcutaneously into the left front armpit of the mice. At the beginning of the experiment, mice were randomly divided into four groups: group 1 animals received injections of vehicle with 5% of a solution containing 70% sorbitol, 25% tween 80 and 5% water (*n* = 10); group 2 animals received injections of piplartine (60 μmol/kg, *n* = 10); group 3 animals received injections of the complex **1** at 15 μmol/kg (*n* = 10); and group 4 animals received injections of the complex **2** at 15 μmol/kg (*n* = 11). Treatments were initiated 1 day after the cancer cell injection. The animals were treated intraperitoneally (200 μL per animal) once a day for 15 consecutive days. One day after the end of the treatment, the animals were anesthetized, and peripheral blood samples were collected from the brachial artery. Animals were euthanized by anesthetic overdose, and tumors were excised and weighed.

#### Toxicological Aspects

Mice were weighed at the beginning and at the end of the experiment. All animals were observed for toxicity signs throughout the whole study. Hematological analysis was performed by light microscopy in blood samples. Livers, kidneys, lungs, and hearts were removed, weighed and examined for any signs of gross lesions, color changes, and/or hemorrhages. After gross macroscopic examination, the tumors, livers, kidneys, lungs, and hearts were fixed in 4% formalin buffer and embedded in paraffin. Tissue sections were stained with hematoxylin/eosin staining, and a pathologist performed the histological analyses under optical microscopy.

### Statistical Analysis

Data are presented as mean ± S.E.M. Differences between experimental groups were compared using analysis of variance (ANOVA) followed by Student–Newman–Keuls test (*p* < 0.05). All statistical analyses were performed using GraphPad Prism (Intuitive Software for Science, San Diego, CA, USA).

## Results

### Ruthenium Complexes With Piplartine Cause Apoptosis Through MAPK Signaling by a p53-Dependent Pathway in HCT116 Cells

**Figure 2:**
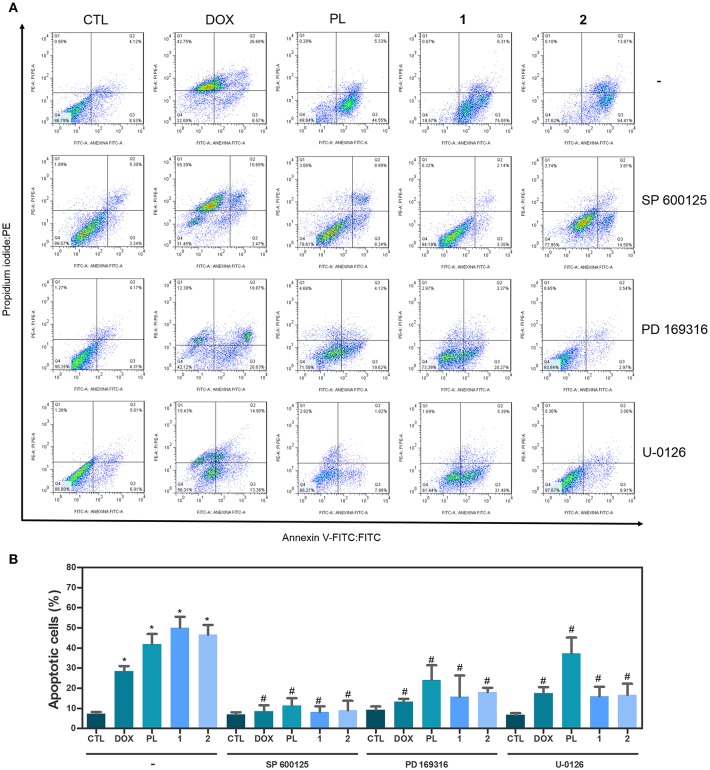
Effect of JNK/SAPK inhibitor (SP 600125), p38 MAPK inhibitor (PD 169316), and MEK inhibitor (U-0126) in the apoptosis induced by ruthenium complexes with piplartine in HCT116 cells, as determined by flow cytometry using Annexin V-FITC/PI staining. **(A)** Representative flow cytometry dot plots showing percentage of cells in viable (annexin V-FITC negative and PI negative cells), early apoptotic (annexin V-FITC positive, but PI negative cells), late apoptotic (annexin V-FITC positive and PI positive cells), and necrotic stages (PI positive, but annexin V-FITC negative cells). **(B)** Quantification of apoptotic HCT116 cells (annexin V-FITC positive cells). For protection assays, cells were pretreated for 2 h with 5 μM SP 600125, 5 μM PD 169316„ or 5 μM U-0126, and then incubated with ruthenium complexes at established concentration (2.5 μM for complex **1** and 5 μM for complex **2**) for 48 h. Negative control was treated with the vehicle (0.1% of a solution containing 70% sorbitol, 25% tween 80 and 5% water) used for diluting the complexes tested. Doxorubicin (1 μM) and piplartine (10 μM) were used as the positive controls. Data are presented as mean ± S.E.M. of at least three independent experiments performed in duplicate. Ten thousand events were evaluated per experiment, and cellular debris was omitted from the analysis. ^*^P < 0.05 compared with negative control by ANOVA, followed by Student-Newman-Keuls test. ^#^*P* < 0.05 compared with respective treatment without inhibitor by ANOVA, followed by Student-Newman-Keuls test.

Apoptotic cell death was quantified by annexin-V/PI double staining using flow cytometry in HCT116 cells after treatment with ruthenium complexes with piplartine at established concentrations (2.5 μM for complex **1** and 5 μM for complex **2**) after 48 h of incubation. Additionally, since mitogen-activated protein kinase (MAPK) signaling play an essential role in apoptotic cell death, the role of the three main MAPK families, Jun N-terminal kinase/stress activated protein kinase (JNK/SAPK), p38 MAPK, and extracellular signal-regulated kinase (ERK) were investigated. Then, we measured complexes-induced apoptosis in HCT116 cells co-treated with JNK/SAPK inhibitor (SP600125), p38 MAPK inhibitor (PD169316), and MEK inhibitor (U-0126, which inhibits the activation of ERK1/2). Both complexes significantly increased percentage of apoptotic cells, and co-treatment with JNK/SAPK, p38 MAPK, and MEK inhibitors significantly reduced complexes-induced apoptosis in HCT116 cells (Figure 2).

**Figure 3:**
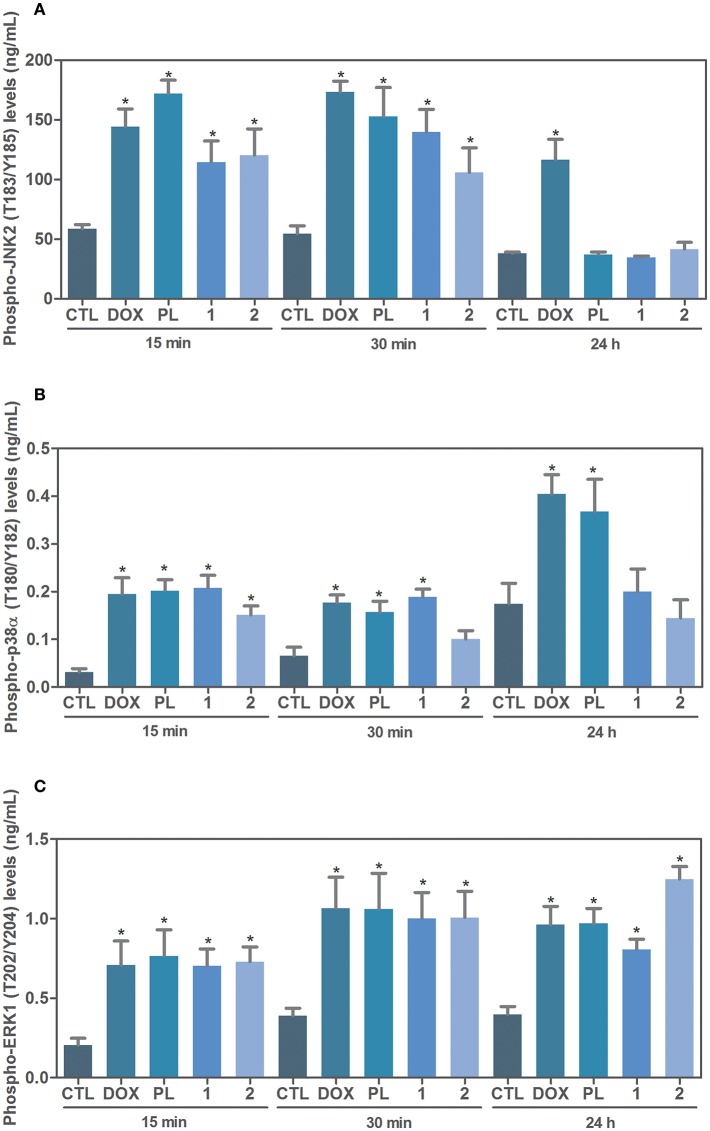
Effect of ruthenium complexes with piplartine in phospho-JNK2 (T183/Y185), phospho-p38a (T180/Y182), and phospho-ERK1 (T202/Y204) levels, as determined by phospho-specific ELISA in HCT116 cells treated with ruthenium complexes at established concentration (2.5 μM for complex **1** and 5 μM for complex **2**) for an acute (15 or 30 min) or prolonged (24 h) incubation. **(A)** Quantification of phospho-JNK2 (T183/Y185) levels. **(B)** Quantification of phospho-p38α (T180/Y182) levels. **(C)** Quantification of phospho-ERK1 (T202/Y204) levels. Negative control was treated with the vehicle (0.1% of a solution containing 70% sorbitol, 25% tween 80 and 5% water) used for diluting the complexes tested. Doxorubicin (1 μM) and piplartine (10 μM) were used as positive controls. Data are presented as mean ± S.E.M. of three independent experiments performed in duplicate. ^*^P < 0.05 compared with negative control by ANOVA, followed by Student-Newman-Keuls test.

Next, the effect of ruthenium complexes with piplartine in phospho-JNK2 (T183/Y185), phospho-p38α (T180/Y182), and phospho-ERK1 (T202/Y204) levels was determined by phospho-specific ELISA in complexes-treated HCT116 cells for an acute (15 or 30 min) or prolonged (24 h) incubation. A significant increase in phospho-JNK2 (T183/Y185) and phospho-p38α (T180/Y182) expressions was observed in complexes-treated cells after 15 and 30 min of incubation (Figures 3A,B). Similarly, a significant increase in phospho-ERK1 (T202/Y204) expression was observed in complexes-treated cells after 15 and 30 min and 24 h of incubation (Figure 3C). Piplartine also increased phospho-JNK2 (T183/Y185) expression after 15 and 30 min of incubation and augmented phospho-ERK1 (T202/Y204) and phospho-p38α (T180/Y182) expressions after 15 and 30 min and 24 h of incubation.

**Figure 4:**
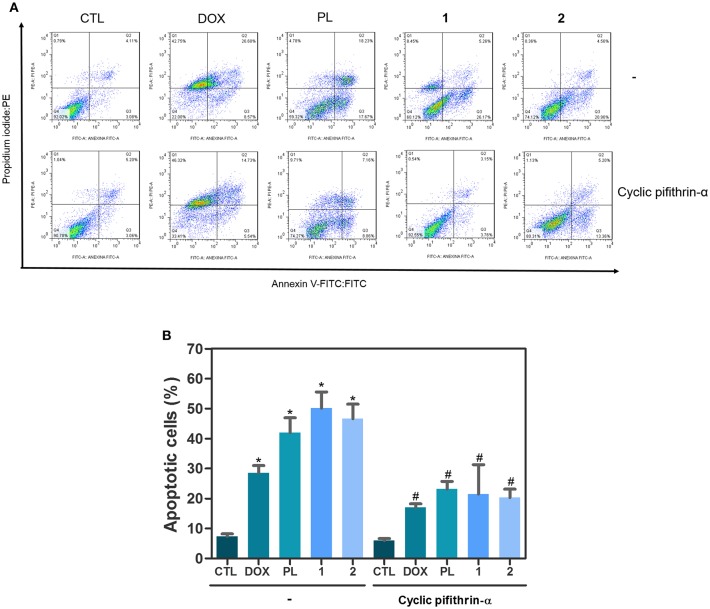
Effect of p53 inhibitor (cyclic pifithrin-α) in apoptosis induced by ruthenium complexes with piplartine in HCT116 cells, as determined by flow cytometry using Annexin V-FITC/PI staining. **(A)** Representative flow cytometry dot plots showing percentage of cells in viable (annexin V-FITC negative and PI negative cells), early apoptotic (annexin V-FITC positive, but PI negative cells), late apoptotic (annexin V-FITC positive and PI positive cells), and necrotic stages (PI positive, but annexin V-FITC negative cells). **(B)** Quantification of apoptotic HCT116 cells (annexin V-FITC positive cells). For protection assays, cells were pretreated for 2 h with 10 μM cyclic pifithrin-α and then incubated with ruthenium complexes at established concentration (2.5 μM for complex **1** and 5 μM for complex **2**) for 48 h. Negative control was treated with the vehicle (0.1% of a solution containing 70% sorbitol, 25% tween 80 and 5% water) used for diluting the complexes tested. Doxorubicin (1 μM) and piplartine (10 μM) were used as positive controls. Data are presented as mean ± S.E.M. of three independent experiments performed in duplicate. Ten thousand events were evaluated per experiment, and cellular debris was omitted from the analysis. ^*^P < 0.05 compared with negative control by ANOVA, followed by Student Newman-Keuls Test. ^#^*P* < 0.05 compared with respective treatment without inhibitor by ANOVA, followed by Student Newman-Keuls Test.

**Figure 5:**
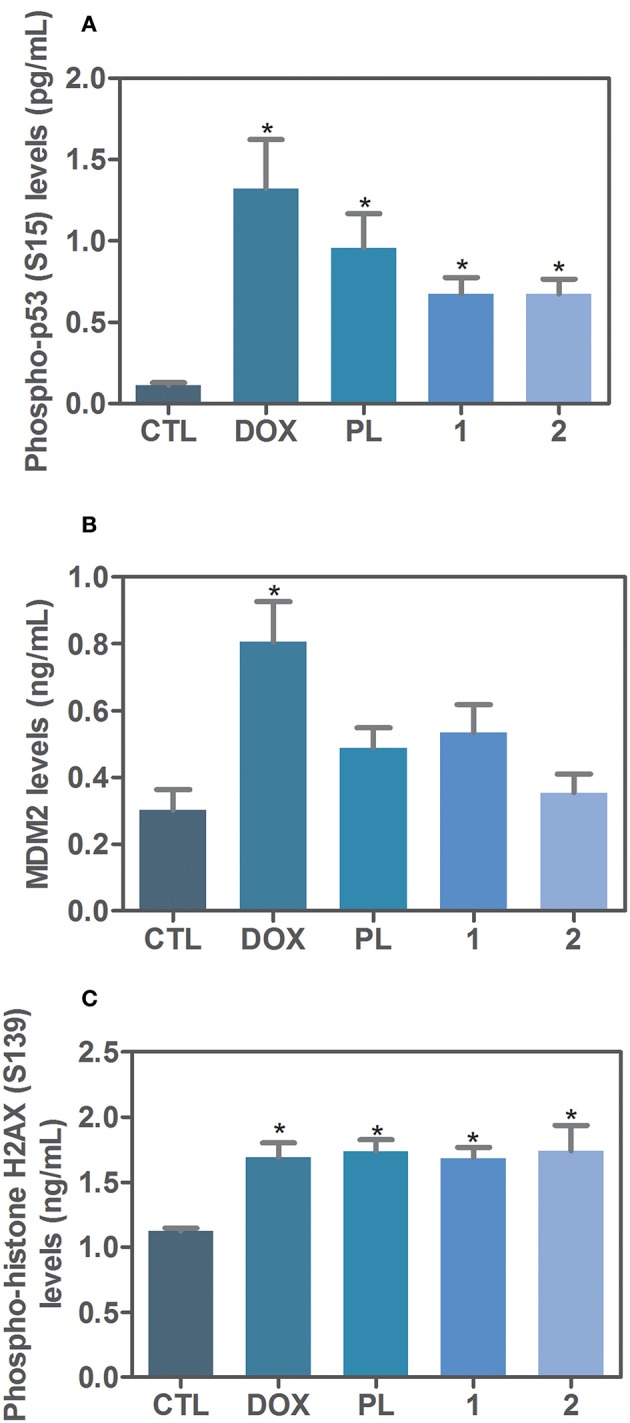
Effect of ruthenium complexes with piplartine in phospho-p53 (S15), MDM2 and phospho-histone H2AX (S139) levels, as determined by phospho-specific ELISA in HCT116 cells treated with ruthenium complexes at established concentration (2.5 μM for complex **1** and 5 μM for complex **2**) for 24 h incubation. **(A)** Quantification of phospho-p53 (S15) levels. **(B)** Quantification of MDM2 levels. **(C)** Quantification of phospho-histone H2AX (S139) levels. Negative control was treated with the vehicle (0.1% of a solution containing 70% sorbitol, 25% tween 80 and 5% water) used for diluting the complexes tested. Doxorubicin (1 μM) and piplartine (10 μM) were used as positive controls. Data are presented as mean ± S.E.M. of three independent experiments performed in duplicate. ^*^P < 0.05 compared with negative control by ANOVA, followed by Student-Newman-Keuls test.

Due the functional interactions between p53 and MAPK signaling pathways, we evaluated the role of the activation of p53 signaling in complexes-induced apoptosis in HCT116 cells. We observed that co-treatment with a p53 inhibitor (cyclic pifithrin-α) significantly reduced complexes-induced apoptosis in HCT116 cells (Figure 4). Moreover, we quantified phospho-p53 (S15), MDM2 and phospho-histone H2AX (S139) expressions in complexes-treated HCT116 cells after 24 h of incubation. Interesting, treatment with ruthenium complexes significantly increased phospho-p53 (S15), and phospho-histone H2AX (S139) expressions (Figure 5).

### Ruthenium Complexes With Piplartine Reduce HCT116 Cell Growth in a Xenograft Model

**Figure 6:**
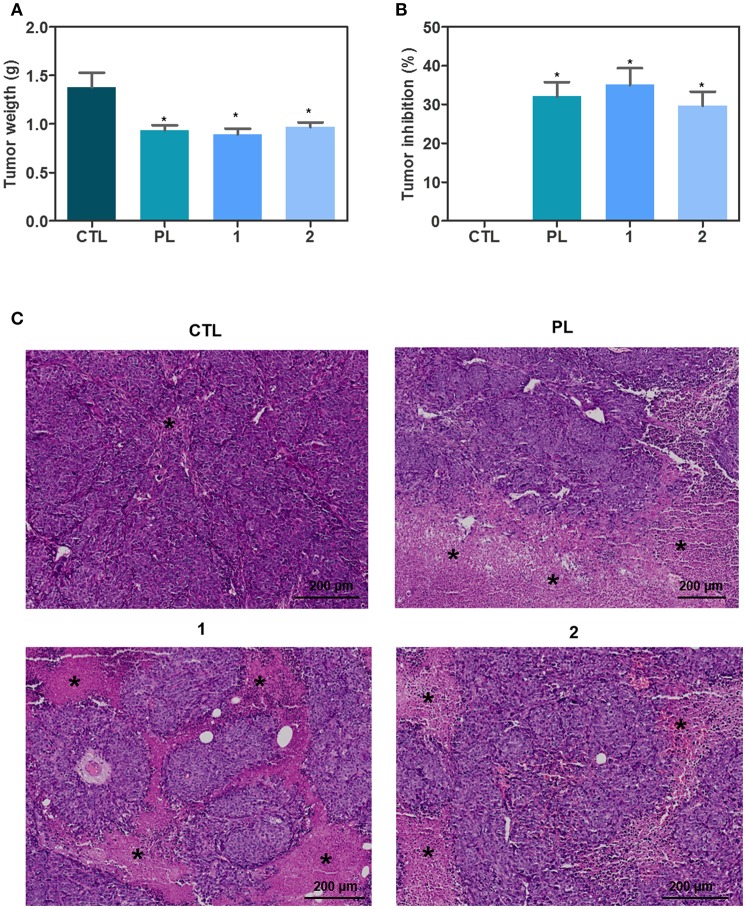
In vivo antitumor activity of ruthenium complexes with piplartine in C.B-17 SCID mice with HCT116 cell xenografts. **(A)** Quantification of tumor weight (g). **(B)** Quantification of tumor inhibition (%). Data are presented as mean ± S.E.M. of 10–11 animals. ^*^P < 0.05 compared with negative control by ANOVA, followed by Student-Newman-Keuls test. **(C)** Representative histological analysis of the tumors stained with hematoxylin and eosin and analyzed by optical microscope. The asterisks represent areas with tumor necrosis. The treatments were initiated 1 day after the cancer cell injection. Animals were treated intraperitoneally once a day for 15 consecutive days. Negative control (CTL) was treated with the vehicle (5% of a solution containing 70% sorbitol, 25% tween 80 and 5% water) used for diluting the complexes. Piplartine (PL, 60 μmol/kg) was used as positive control.

Concerning *in vivo* action of ruthenium complexes with piplartine, anti-colon cancer effect was evaluated in C.B-17 SCID mice engrafted with HCT116 cells, and animals were treated by intraperitoneal injections for 15 consecutive days with complex **1** at dose of 15 μmol/kg/day, complex **2** at dose of 15 μmol/kg/day, and piplartine at dose of 60 μmol/kg/day. Both complexes were able to inhibit statistically significant HCT116 cell growth in xenograft model (Figures 6A, **B**). On 16th day, the mean of tumor mass weight of negative control group was 1.38 ± 0.15 g. In animals treated with complex **1**, the mean of tumor mass weights was 0.89 ± 0.06 g, while was 0.97 ± 0.05 g in animals treated with complex **2**. Piplartine-treated animals showed a mean of tumor mass weights of 0.94 ± 0.05 g. Tumor mass inhibition rates were 35.06, 29.71, and 32.03% for complex **1** (15 μmol/kg/day), complex **2** (15 μmol/kg/day), and piplartine (60 μmol/kg/day), respectively. In the histological analysis, all groups exhibited a predominant poorly differentiated adenocarcinoma with solid growth pattern with extensive areas of tumor necrosis for groups treated with piplartine and ruthenium complexes (Figure 6C).

With regards to the toxicological aspects, body and organ (liver, kidney, lung, and heart) weights, and hematological analysis were assessed in all mice after the end of treatment. No significant alterations were observed in body weight neither in liver, kidney, lung, or heart wet weight of any group (*P* > 0.05) (data not shown). In addition, all hematological parameters analyzed in mice treated the ruthenium complexes were similar to those of naïve controls (*P* > 0.05) (data not shown).

Morphological analyses of liver, kidneys, lungs, and hearts in all groups were performed. Histopathological analyses of lungs revealed significant inflammation predominantly of mononuclear cells, edema, congestion and hemorrhage, ranging mild to severe. It is important to note that these histopathological alterations were more pronounced in negative control, piplartine and complex **2** groups than in complex **1** group. The architecture of parenchyma was partially maintained in all groups, observing a thickening of the alveolar septum with decreased airspace, ranging from mild to moderate. In addition, tumor nodules and embolus in lungs were observed only in one animal of negative control group and complex **2**, respectively. In livers, the acinar architecture and centrilobular vein were also preserved in all groups. Focal areas of inflammation and coagulation necrosis were observed in negative control, complex **1** and complex **2**. Other findings, such as congestion and hydropic degeneration were found in all groups, ranging from mild to moderate. In kidneys, tissue architecture was preserved in all experimental groups. Histopathological changes included vascular congestion and thickening of basal membrane of renal glomerulus with decreased urinary space were observed in all kidneys, ranging from mild to moderate. Histopathological analysis of animal hearts did not show alterations in any group.

## Discussion

Two ruthenium complexes with piplartine recently designed and synthesized were shown to be potential anticancer agents that target oxidative stress and cause cell death in cancer cells with higher potency than metal-free piplartine (31). Herein, we demonstrated the intracellular processes modulated by these ruthenium complexes, involving MAPK (JNK, p38 MAPK, and ERK1/2) signaling by a p53-dependent pathway, that trigger apoptosis in HCT116 cells. More importantly, we showed here that these two ruthenium complexes inhibit tumor development in a xenograft mouse model more potently than piplartine.

All three JNK/SAPK (JNK-1, JNK-2, and JNK-3) can trigger the apoptotic pathway by stimulating expression of pro-apoptotic genes through activation of specific transcription factors, including c-Jun, p53, and p73 (32). In this work, co-treatment with a JNK1-3 inhibitor (SP 600125) reduced ruthenium complexes-induced apoptosis in HCT116 cells, which was confirmed by quantification of levels of phosphorylation of JNK2 (T183/Y185), indicating JNK-mediated apoptosis. In fact, D'Sousa Costa et al. (31) found that these complexes up-regulated MAPK-related genes in HCT116 cells. Piplartine has been previously shown to inhibit cell proliferation and cause apoptosis in human melanoma cells via ROS and JNKs pathways (33). Moreover, piplartine increased the phosphorylation of p38 and JNK in bone marrow mononuclear cells from patients with myeloid leukemias, and co-treatment with specific p38 or JNK inhibitors partially reversed piplartine-induced processes, such as ROS production and apoptotic/autophagic signaling activation (34). Piplartine also induced ROS accumulation, leading to cholangiocarcinoma cell apoptosis via activation of JNK/ERK pathway (20). Interestingly, a ruthenium methylimidazole complex caused ROS accumulation and ROS-mediated DNA damage by MAPK (JNK and p38 MAPK) and AKT signaling pathways in lung carcinoma A549 cells (35). Altogether, these findings support the role of JNK pathway in pro-apoptotic mechanism triggered by ruthenium complexes with piplartine.

Four p38 MAPK isoforms have been identified: p38α, p38β, p38γ and p38δ, being p38α and p38β the most studied isoforms. This MAPK signaling is activated in response to UV damage, oxidative stress, exposure to DNA damaging agents and growth factors and cytokines. Its activation modulates a wide variety of cellular functions, such as protein kinases, phosphatases, cell-cycle regulators and transcription factors, including p53 (36). In order to evaluate whether the p38 MAPK pathway is involved in ruthenium complexes-induced apoptosis in HCT116 cells, we co-treated the cells with a p38 MAPK inhibitor (PD 169316) and ruthenium complexes. Co-treatment with p38 MAPK inhibitor reduced the complexes-induced apoptosis in HCT116 cells, and was also confirmed by quantification of the levels of phosphorylation of p38α (T180/Y182), indicating p38 MAPK-mediated apoptosis. Wang et al. (37) reported that piplartine induces apoptosis and autophagy in leukemic cells through targeting the PI3K/Akt/mTOR and p38 signaling pathways. Moreover, a platinum complex with piplartine caused ROS-mediated apoptosis by ERK1/2/p38 pathway in human acute promyelocytic leukemia HL-60 cells (17).

ERK1/2 MAPK signaling belongs to the RAS-regulated RAF-MEK-ERK signaling pathway, and activation of ERK1/2 leads phosphorylation of more than 200 substrates (38). Therefore, the consequences of ERK1/2 activation are diverse that include some apparently contradictory biological responses such as cell cycle progression or cell cycle arrest and cell survival or cell death. The type of cellular response is determined by some factors, such as the duration and intensity of ERK1/2 activation, co-activation of other pathways and subcellular distribution of ERK1/2 (38). Herein, co-treatment with a MEK inhibitor (U-0126, which inhibits the activation of ERK1/2) reduced ruthenium complexes-induced apoptosis in HCT116 cells, and an increased phosphorylation of ERK1 (T202/Y204) was demonstrated, indicating ERK1/2-mediated apoptosis. The ruthenium complex with xanthoxylin was previously reported to induce S-phase arrest and cause ERK1/2-mediated apoptosis in HepG2 cells through a p53-independent pathway (25).

The tumor suppressor p53 has been shown to induce cell-cycle arrest, promote DNA repair or induce apoptotic cell death in response to cellular stress. The p53 signaling activation is induced by several cellular stress signals, including DNA damage and oxidative stress (36). In addition, DNA damage can be monitored by quantification of phosphorylation of histone H2AX (γH2AX) that is an early sign of DNA damage. Herein, both ruthenium complexes with piplartine were able to increase phosphorylation of the histone H2AX (S139), and co-treatment with a p53 inhibitor (cyclic pifithrin-α) reduced complexes-induced apoptosis in HCT116 cells, which was confirmed by increasing in levels of phosphorylation of p53 (S15), indicating p53-dependent apoptosis. Corroborating with these results, D'Sousa Costa et al. (31) observed that TP53 gene was up-regulated in HCT116 cells treated with complex **1**. Since the p53 protein can functionally interact with MAPK pathways, including JNK/SAPK, the p38 MAPK, and the ERK1/2, these results corroborate the apoptosis induction through MAPK signaling by a p53-dependent pathway in complexes-treated HCT116 cells.

Human tumor xenograft mouse model is one of the most widely used models to evaluate *in vivo* antitumor effect of new compounds. It remain human tumor cell heterogeneity, madding possible predict the drug response in human patient as well allows a fast analysis of human tumor response *in vivo* protocols (39, 40). Therefore, we also investigated *in vivo* anti-colon cancer of ruthenium complexes with piplartine in C.B-17 SCID mice xenografted with HCT116 cells. These complexes were able to inhibit tumor growth with higher potency that piplartine, since complexes tested at doses of 15 μmol/kg/day showed similar efficacy observed to piplartine tested at dose of 60 μmol/kg/day. No significant changes were observed in body and organs weights neither hematological parameters of any group, indicating low toxicity of these complexes. These *in vivo* results corroborate the potential use of piplartine-based compounds for colon cancer treatment previously described. Piplartine and a N-heteroaromatic ring-based analog were reported to repress tumor growth in HCT116 xenograft mouse model (40). The ruthenium complex with xanthoxylin was previously reported to inhibit the development of HepG2 cells in xenograft model (25). Moreover, a ruthenium complex inhibited dose-dependently the growth of human hepatocarcinoma cells BEL-7402 in xenotransplanted mice (41).

**Figure 7:**
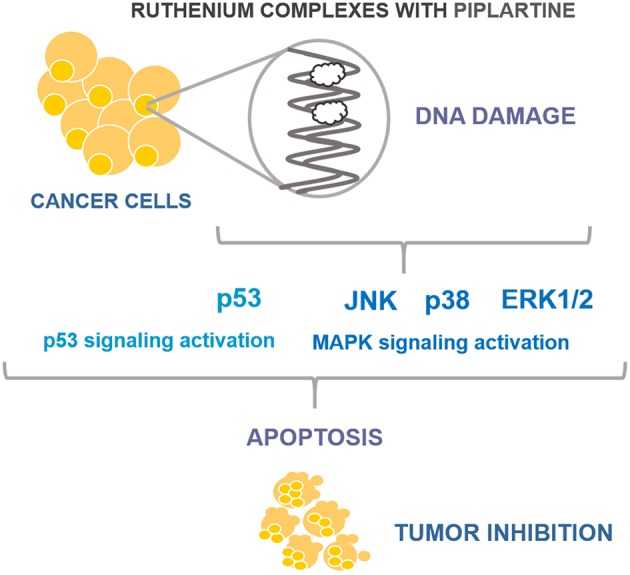
Summary of the mechanisms of action of ruthenium complexes with piplartine in HCT116 cells.

In conclusion, we showed here that ruthenium complexes with piplartine cause apoptosis through MAPK signaling by a p53-dependent pathway in HCT116 cells (Figure 7), and are able to inhibit HCT116 cell growth in xenograft model with higher potency than piplartine alone. These data indicate these complexes as promising new anti-colon cancer drugs candidates.

## Ethics Statement

The institutional Animal Ethics Committee of Gonçalo Moniz Institute approved the experimental protocol (number 06/2015).

## Author Contributions

IB, MS, JN, AB, and DB conceived and designed the experiments. IB, SS, LS, JN, RD, CS, and CR performed the *in vitro* and *in vivo* experiments. IB, SS, CR, AB, and DB analyzed the data: CR, MS, AB, and DB contributed reagents, materials, and analysis tools. DB wrote the paper. All authors read and approved the final manuscript.

### Conflict of Interest Statement

The authors declare that the research was conducted in the absence of any commercial or financial relationships that could be construed as a potential conflict of interest.
